# Status and influencing factors of elder neglect by geriatric nursing assistants in Chinese nursing homes: a cross-sectional survey

**DOI:** 10.3389/fmed.2023.1273289

**Published:** 2023-10-25

**Authors:** Jing Wang, Zhihua Yang, Ya Li, Ruijuan Ma, Liping Zhang, Yage Du, Haoying Dou

**Affiliations:** ^1^School of Nursing, Tianjin University of Traditional Chinese Medicine, Tianjin, China; ^2^Affiliated Cancer Hospital and Institute of Guangzhou Medical University, Guangzhou, China; ^3^School of Media and Communications, Urumqi Vocational University, Urumqi, China; ^4^Shandong Provincial Hospital Affiliated to Shandong First Medical University, Jinan, China; ^5^School of Nursing, Peking University, Beijing, China

**Keywords:** elder neglect, geriatric nursing assistants, proactive personality, self-efficacy, nursing homes, older adults

## Abstract

**Background:**

In nursing homes, elder neglect has come to the forefront. Currently, few studies have examined the impact of personal and organizational factors of geriatric nursing assistants on elder neglect. From the perspective of geriatric nursing assistants, this study aims to explore the current situation and influencing factors of elder neglect in Chinese nursing homes.

**Methods:**

A convenience sampling method was used to recruit 412 geriatric nursing assistants from 50 nursing homes in China. Participants were surveyed using a demographic questionnaire, the Elder Neglect Scale for Geriatric Nursing Assistants, the General Self-Efficacy Scale (GSES), and the Proactive Personality Scale (PPS). Spearman correlation analysis and multiple linear regression were used to analyze the factors influencing elder neglect.

**Results:**

Geriatric nursing assistants scored a median of 74 out of 85 on the Elder Neglect Scale. Multiple linear regression analyses showed that the main personal factors influencing geriatric nursing assistants’ elder neglect were general self-efficacy (*β* = 0.312), proactive personality (*β* = 0.180), and advanced qualification (*β* = 0.084), while the main organizational factors included monthly salary ≤ 1,900 RMB (*β* = −0.256), no regular training after induction (*β* = −0.253), and the number of days off per month (3–4 days off *β* = 0.192, ≥ 5 days off *β* = 0.101).

**Conclusion:**

Although geriatric nursing assistants are at low levels of elder neglect, it remains a cause for concern. Among the personal factors, geriatric nursing assistants who possessed proactive personalities, high self-efficacy and advanced qualifications, exhibited low levels of elder neglect. Among the organizational factors, those who possessed a high number of days off per month portrayed low levels of elder neglect. Conversely, those who received low monthly salaries and no regular training after induction portrayed high levels of elder neglect. To reduce the risk of elder neglect, nursing homes should give due consideration to candidates’ self-efficacy and proactive personality traits when recruiting, and focus on fostering these personality traits in their employees during their work. In addition, strengthening regular training for geriatric nursing assistants, optimizing the salary structure, and arranging rest days in a reasonable manner are also necessary measures.

## 1. Introduction

### 1.1. Background

Elder abuse is a public health and social issue of global concern. The World Health Organization (WHO) divided elder abuse into five categories, one of which is elder neglect (1). It is described as a caregiver’s failure to provide basic or essential services to an older adult or protect them from injury (2). Elder neglect could be detrimental to physical and mental health. Older adults who experience neglect have greater mortality rates and readmissions than those who do not (3).

Elder neglect is probably more prevalent in institutional settings than in community settings. According to two meta-analyses, the prevalence of elder neglect was 11.6% in institutional settings and 4.2% in community settings (4, 5). It may be because older adults in institutional settings are frailer and at higher risk of neglect. Elderly neglect is affected by a combination of psychosocial, economic, and cultural factors. Its prevalence varies widely depending on the area, the population, and the survey instrument. In a scoping review, the prevalence of neglect reported by older adults in nursing homes ranged from 16–87%, and the prevalence reported by staff ranged from 1–77% (6). In Norway, a study surveyed 3,693 staff in 100 nursing homes, and 46.9% admitted to at least one case of elder neglect in the past year (7). As the population of older adults increases, the prevalence of elder neglect will likely continue to rise. However, older adults may fail to report neglect in a timely manner due to fear, cognition problems, or a taboo of the subject (8). Therefore, the incidence of elder neglect may be seriously underestimated.

What elements play a role in elder neglect? The literature on elder neglect in nursing homes focused on three main factors: resident characteristics, staff characteristics, and organizational environment. Older adults’ poor health, extreme caregiver stress, or poor environmental conditions may lead to elder neglect (6, 7, 9). Table 1 showed the adverse factors affecting elder neglect.

**Table 1:** Adverse factors affecting elder neglect.

		Adverse factors
Personal factors	Resident	Lack of financial resources, increased dependence on caregivers, depression, anxiety, and dementia
	Staff	Inappropriate staff arrangements, low compensation, insufficient training and supervision, bad working conditions, and significant staff turnover
Organizational factors		Lack of social support, poor institutional environment, and disorganized management

### 1.2. Rationale for the study

As the number of older adults is increasing, the aging population has become a worldwide challenge. The Chinese older adult population is particularly enormous, and the aging of the population is rapidly speeding up. According to China’s seventh national population census, the number of people aged 60 and above in 2020 was 264 million, accounting for 18.70% of the total population (10), and it is expected that the number of people aged 60 and above will reach 498 million by 2050 (11). At present, over 2.1 million older adults live in institutions in China. Institutional care has become a primary way of retirement enjoyment for older adults. The older adults living in nursing homes are generally older, mainly aged 80 years and over, mostly widowed or divorced women with adult children (12). Most of them are sick and depend on others for help in their daily lives. They receive round-the-clock from employed professional geriatric nursing assistants working in shifts. The working hours of geriatric nursing assistants include 8-h/12-h shifts (i.e., 5 days on, 2 days off) and 24-h shifts (i.e., 1 day on, 1 day off). Even on national holidays, they have to continue working in shifts and rarely take a break. The high labor intensity may make it impossible for them to care well for every older adult, potentially increasing the risk of elder neglect. The same situation exists in other countries. In Italian nursing homes, most nurses work over 10 h a day, but not more than 24 h (13). With the increasing number of nursing home residents with severe clinical illnesses that require specific medical care services, a mismatch between the high workload on nurses and staffing results in a lack of time to care for the residents and, ultimately, missed care.

Self-efficacy is described as a person’s conviction in their talents and abilities to perform a task satisfactorily (14). A study found that geriatric nursing assistants with high self-efficacy were more focused on their work and produced high-quality care (15). Therefore, employees who have higher self-efficacy are more likely to do their jobs well and perform better.

The proactive personality is a stable personality disposition in which people engage in proactive acts to impact their surroundings (16). A proactive personality helps employees to clarify their responsibilities and integrate into the team by influencing their ability to adapt to the environment (17). A study showed that a proactive personality will affect nurses’ competence and work engagement (18). Consequently, we suggest that the self-efficacy and personality traits of geriatric nursing assistants would influence their attitudes and quality of work. However, no research has been done in nursing homes to investigate the relationship between elder neglect, geriatric nursing assistants’ personality, and self-efficacy.

### 1.3. Study questions or hypotheses

Previous research has explored the prevalence, risk factors, prevention, and intervention of elder neglect in some countries (19). However, at present, there are no specialized agencies or staff to investigate elder neglect in China. In addition, the applicability of most of the assessment tools on elder neglect in Chinese nursing homes is unknown due to the vast differences in cultural backgrounds and conditions of application. It is regretted that there has been little research on the incidence of elder neglect in China’s nursing homes.

This study aims to investigate the level of elder neglect in Chinese nursing homes and its association with geriatric nursing assistants’ personal and organizational factors, such as compensation, training, supervision, and working conditions (H1). Additionally, we hypothesize that high levels of self-efficacy and a proactive personality among geriatric nursing assistants will reduce the level of elder neglect (H2). We expect that the results of this study will help nursing homes more clearly recognize the potential impact of geriatric nursing assistants on elder neglect and provide a basis for the development of prevention and intervention policies to enhance the nursing care service system, reduce the level of elder neglect in nursing homes and further improve the quality of life of older adults.

## 2. Methods

### 2.1. Study design

This survey used a cross-sectional design, in which nursing homes were conveniently selected from the list of registered institutions on the official website of the Ministry of Civil Affairs. The data was collected between July 2020 and September 2020. The research report was written following the Strengthening the Reporting of Observational Studies in Epidemiology (STROBE) checklist.

### 2.2. Setting and participants

#### 2.2.1. Participants

The inclusion criteria were: (1) directly responsible for caring for older adults, (2) caring for an older adult in a nursing home for at least 3 months, (3) having the basic reading, writing, or understanding ability, and (4) can voluntarily provide informed consent. The exclusion criteria were: (1) not on duty during the study period.

#### 2.2.2. Sample size

The sample size should be 5–10 times the number of variables, according to the Kendall sample estimate method. The number of variables included in this survey was 37. As a result, we determined that the sample size for this study should be 185–370. In addition, we statistically considered that there may be a 20% questionnaire failure rate, and 232–463 questionnaires should be finally distributed.

#### 2.2.3. Recruitment, exposure, and follow-up periods

Geriatric nursing assistants were recruited from nursing homes in Henan, Hebei, and Shandong provinces, China. The researchers obtained the contact information of the nursing homes in the three regions from the official website of the Ministry of Civil Affairs and then obtained the consent and support of 50 nursing home directors through telephone or email contact. The data was collected between July 2020 and September 2020.

### 2.3. Data collection

Before the survey, all researchers received uniform training to ensure consistency of study results. The researchers were familiar with the content of the questionnaires and standardized the instructions’ terminology after training to avoid vague and misleading statements. The researchers arrived at the nursing homes on the specified dates and explained the purpose, significance, cooperation, and confidentiality of the study to the eligible geriatric nursing assistants. All eligible geriatric nursing assistants had equal rights to participate in this study. Only eligible geriatric nursing assistants who have given fully informed consent and signed the informed consent form would participate in the survey and answer the questions. For geriatric nursing assistants who were unable to complete the questionnaire on their own due to low literacy, the researchers read the information in the questionnaire to the participants in a neutral tone and recorded their answers. Geriatric nursing assistants were not obligated to participate in any way, and their wishes would be fully respected throughout the survey process.

Additionally, appropriate quality control measures are taken to ensure the data’s authenticity, reliability and completeness. First, the researchers selected elderly caregivers according to strict criteria and distributed the questionnaires in several nursing homes in several regions to minimize selection bias caused by convenience sampling. Second, given the sensitivity of the research questions, the anonymity and confidentiality of the survey were declared to participants before completing the questionnaire to eliminate their concerns. In addition, it was necessary to ensure that no one else was present during the investigation to minimize self-reporting bias. Finally, the questionnaires were distributed and collected on the spot, and participants were asked to fill in any missing entries.

### 2.4. Measures

#### 2.4.1. Demographic questionnaire

In this study, a self-designed demographic questionnaire was used. There were two sections to the questionnaire. The first section contains demographic data on geriatric nursing assistants. It mainly includes the geriatric nursing assistant’s gender, age, household location, marital status, and education level. The second section is about the geriatric nursing assistants’ work situation. It mainly includes whether they are licensed to work, working years, salary, nature of employment, number of older adults to care for per day, and working hours, etc.

#### 2.4.2. Elder neglect scale for geriatric nursing assistants

The scale is a self-assessment scale developed by Du et al. (20) using situational theory as a theoretical basis. To test the scale, Du et al. administered it to 407 geriatric nursing assistants, from which 30 were selected for retesting. The final scale includes four dimensions of physical neglect (5 items), psychological neglect (4 items), economic neglect (3 items), and medical neglect (5 items), with a total of 17 items. A sample item was, “Do you remind or help older adults to wear seasonal clothing?” The scale uses a Likert scale from 0 to 5, with each item rated from 0 (never or not applicable) to 5 (always). The total score ranged from 0 to 85. The lower the score, the higher the level of geriatric nursing assistants’ neglect of the older adult. The Cronbach’s alpha coefficient for this scale is 0.88. The retest reliability for the scale was 0.94. The content validity of the scale was 0.99. The Cronbach’s alpha value of this survey was determined to be 0.83.

#### 2.4.3. General self-efficacy scale (GSES)

The GSES was used to measure geriatric nursing assistants’ self-efficacy. It was developed by German scholars Schwarzer et al. (21). based on self-efficacy theory and was translated and adapted into Chinese (22). A sample item was “It is easy for me to stick to my aims and accomplish my goals.” The scale was a unidimensional scale consisting of ten items that responded using a 4-point Likert scale from 1 (not at all true) to 4 (completely true). The total score range was 10 to 40. The higher the score, the higher the level of self-efficacy. The validity and reliability test results of the scale were good, with an internal consistency of 0.91 (22). The scale is now universally applicable among various populations in China. In the present study, Cronbach’s alpha coefficient was 0.82.

#### 2.4.4. Proactive personality scale (PPS)

The Proactive Personality Scale (23) was applied to assess the proactive personality of geriatric nursing assistants. The scale was based on a streamlined version of Bateman’s 17-item scale (16), with 10 items. A sample item was “I’m always looking for better ways to solve problems.” Geriatric nursing assistants can respond on a five-point Likert scale ranging from 1 (strongly disagree) to 5 (entirely agree). The total score ranged from 10 to 50. The higher the total score, the higher the initiative. The Chinese translation of the scale had good psychometric properties, with a Cronbach’s alpha coefficient of 0.84 (24, 25). This one-dimensional scale was widely used among different populations in China because of its suitability for the Chinese situation and ease of use. Cronbach’s alpha coefficient in this study was 0.74.

### 2.5. Data analysis

SPSS statistical software (version 26.0) was used for all of the analyses. To determine if the data had a normal distribution, the Shapiro–Wilk test was utilized, which showed that the data was nonnormally distributed. Therefore, frequencies and percentages were used for descriptive statistics for categorical data and median and interquartile range (IQR) for continuous variables. For non-normally distributed data, we used the nonparametric test. The Mann–Whitney U test was used for dichotomous variables, and the Kruskal-Wallis test was used for multicategorical variables to determine if there was a significant relationship between the distributions of elder neglect by geriatric nursing assistants’ characteristics. Bonferroni was used for post-hoc comparisons. Correlation analysis was performed using Spearman’s test to evaluate the association of geriatric nursing assistants’ proactive personality, self-efficacy, and elder neglect. Spearman’s correlation coefficient (r_s_) was calculated, with r_s_ > 0.5 as a strong correlation, 0.3 to 0.5 as a moderate correlation, and < 0.3 as a weak correlation (26). Potential confounding variables involved in this study included geriatric nursing assistants’ gender, age, residence, marital status, education, and the nature of the institution and employment they worked for. To control for confounding variables, variables showing statistically significant differences (*p* < 0.05) in the results of univariate and correlation analyses were included in stepwise multiple linear regression analyses to identify factors influencing elder neglect. A significance level was set at 0.05. If the entry missing value or invalid answers were greater than or equal to 5%, the questionnaire was excluded. If the missing value of entries was within 5%, the mean value method was used to estimate the average of all response units.

It needs to be explained that the geographic areas investigated in this study are geographically located in North China, and the social environment and cultural backgrounds are similar. Moreover, the majority of the surveyed nursing homes were located in urban areas with less overall regional variation in geriatric nursing assistants. Therefore, subgroup analyses were not performed by province.

### 2.6. Ethical considerations

The Medical Ethics Committee of Tianjin University of Traditional Chinese Medicine evaluated this work and approved it. The researchers went over the study with the subjects in detail and ensured that they did not have any objections to the study. The survey was based on the principle of voluntary participation and was conducted anonymously after participants signed an informed consent form. There were no negative consequences for geriatric nursing assistants refusing to participate in this study. All study participants were informed about the confidentiality of their responses and the preservation of their anonymity.

## 3. Results

A total of 449 questionnaires were distributed, excluding 20 containing incomplete responses and 17 with invalid answers, 412 valid questionnaires were received, with a valid recovery rate of 91.8%. The final sample sources were 152 geriatric nursing assistants from 17 institutions in Henan Province, 102 geriatric nursing assistants from 15 institutions in Hebei Province, and 158 geriatric nursing assistants from 18 institutions in Shandong Province.

### 3.1. Characteristics of geriatric nursing assistants

Of the 412 responses analyzed for our study, 350 (85.0%) were female, 347 (84.2%) were married, 343 (83.3%) lived in cities and towns, and the majority (*n* = 351, 85.2%) were between 41 and 60 years of age. Junior high school, elementary school education level, or below accounted for 73.8% of participants. Most participants were temporary workers (*n* = 240, 58.3%) who worked in private institutions (*n* = 285, 69.2%), with nearly half working for more than 5 years (*n* = 187, 45.4%). And the caregivers’ working hours range from 8 to 24 h per day. 154 (37.4%) provided daily group care for all older adults. 48.3% (*n* = 199) did not take more than 2 days off per month. The majority in our survey sample (90.3%) trained before employment and more than 82% received training after induction. 303 (73.5%) were licensed and 338 (82.0%) hold different levels of qualification certificates. Among all the caregivers, nearly half of the participants had a salary range of 1,901–3,000 RMB (46.8%) and over two-thirds did not have social insurance (83.0%). More demographic information is shown in Table 2.

**Table 2:** Characteristics of participants and univariate analysis of elder neglect scores (*n* = 412).

Variables	*n* (%)	Median (IQR)	*Z*/*H* ^†^	*p*-value	Bonferroni test
Demographics					
Gender			−0.02	0.984	
Male	62 (15.0)	74 (69–79)			
Female	350 (85.0)	74 (67–80)			
Marital status			3.13	0.372	
Single	13 (3.2)	76 (70–82.5)			
Married	347 (84.2)	73 (67–80)			
Divorced	27 (6.6)	76 (70–79)			
Widowed	25 (6.1)	78 (68–80.5)			
Residence			−0.51	0.609	
Cities and towns	343 (83.3)	74 (69–80)			
Rural areas	69 (16.7)	74 (67–80)			
Age			2.77	0.597	
≤ 30 years old	14 (3.4)	75 (65–80.5)			
31–40 years old	21 (5.1)	78 (64.5–80.5)			
41–50 years old	147 (35.7)	75 (68–80)			
51–60 years old	204 (49.5)	74 (68–80)			
≥ 61 years old	26 (6.3)	71 (65.5–75.5)			
Education level			11.18	0.011^*^	a < d
Primary school or below^a^	105 (25.5)	72 (64.5–79)			
Junior high school^b^	199 (48.3)	74 (67–80)			
High school or junior college^c^	90 (21.8)	71.5 (68–80)			
University or above^d^	18 (4.4)	79.5 (75.5–83)			
Work situation					
Nature of institution			18.94	< 0.001^***^	a < b
Public^a^	84 (20.4)	69 (64–76.75)			
Private^b^	285 (69.2)	76 (69.5–80)			
Public-private^c^	43 (10.4)	70 (65–80)			
Nature of employment			23.89	< 0.001^***^	a, c < b
Officially on staff^a^	20 (4.9)	70 (66–72.5)			
Employed on contract^b^	152 (36.9)	78 (70–82)			
Temporary workers^c^	240 (58.3)	72 (67–79)			
Working years			21.52	< 0.001^***^	e < c
< 1 year^a^	34 (8.3)	74 (69.5–81)			
1–3 years^b^	101 (24.5)	75 (64.5–81)			
3–5 years^c^	90 (21.8)	78 (71.5–82)			
5–10 years^d^	112 (27.2)	73.5 (67–79)			
> 10 years^e^	75 (18.2)	70 (66–77)			
Daily working hours			12.17	0.002^**^	c < a,b
8–10 hours^a^	103 (25.0)	77 (68–82)			
11–14 hours^b^	127 (30.8)	76 (69–81)			
15–24 hours^c^	182 (44.2)	72 (66–79)			
Number of older adults cared for per day			4.14	0.388	
Collective care	154 (37.4)	75 (67–81)			
One-to-one service	9 (2.2)	75 (70–85)			
2–4 persons	93 (22.6)	72 (67.5–80.5)			
5–7 persons	72 (17.5)	74 (71.25–79)			
≥ 8 persons	84 (20.4)	74.5 (66–79)			
Days off per month			59.36	< 0.001^***^	a < b, c
0–2 days^a^	199 (48.3)	70 (65–76)			
3–4 days^b^	154 (37.4)	79 (71–82)			
≥ 5 days^c^	59 (14.3)	77 (74–80)			
Whether received pre-service training			−4.99	< 0.001^***^	
Yes	373 (90.3)	75 (68–80)			
No	40 (9.7)	69.5 (61.25–72)			
Whether received regular training after induction			−7.72	< 0.001^***^	
Yes	339 (82.3)	76 (69–81)			
No	73 (17.7)	65 (61.5–71)			
Whether worked with a license			−2.09	0.036^*^	
Yes	303 (73.5)	75 (68–80)			
No	109 (26.5)	73 (64–78)			
Level of qualification certificate			34.26	< 0.001^***^	e < a, b, c, d; a < c
Primary qualification^a^	197 (47.8)	74 (68–79)			
Intermediate qualification^b^	54 (13.1)	74 (69–82.25)			
Advanced qualification^c^	24 (5.8)	80 (77–84)			
Qualified qualification^d^	63 (15.3)	76 (68–80)			
No^e^	74 (18.0)	70 (62–74)			
Monthly salary (RMB)			75.27	< 0.001***	a < b, c, d
≤ 1,900^a^	112 (27.2)	68 (62–72)			
1,901–3,000^b^	193 (46.8)	75 (70–80)			
3,001–4,000^c^	85 (20.6)	79 (73–83)			
4,001–5,000^d^	22 (5.3)	78.5 (72–83)			
Whether had social insurance			−2.46	0.014^*^	
Yes	70 (17.0)	77 (69–82)			
No	342 (83.0)	73 (67–80)			

^*^p-value <0.05; ^**^p-value *<*0.01; ^***^p-value *<*0.001.†Mann Whitney U test/Kruskal-Wallis test.The letters “^a–e^” represent the level of elder neglect by geriatric nursing assistants in the respective subgroups.

### 3.2. Characteristics of the elder neglect scale for geriatric nursing assistants scores

The total score on the Elder Neglect Scale for Geriatric Nursing Assistants ranged between 43 and 85, and the median was 74 (IQR: 68–80). The median scores ranked from highest to lowest were for physical neglect (Median = 24; IQR: 22–25), medical neglect (Median = 23; IQR: 22–24.75), psychological neglect (Median = 17; IQR: 14–19), and economic neglect (Median = 12; IQR: 9–14) (Table 3).

**Table 3:** Characteristics of the scores on scales (*n* = 412).

Variables	Median (IQR)	Min-Max
Total score of elder neglect	74 (68–80)	43–85
Physical neglect	24 (22–25)	10–25
Psychological neglect	17 (14–19)	5–20
Economic neglect	12 (9–14)	3–15
Medical neglect	23 (22–24.75)	12–25
Self-efficacy score	28 (25–32)	10–40
Proactive Personality Score	40 (37–43)	18–50

### 3.3. Univariate analysis of elder neglect scores

Univariate analysis identified statistical differences in the elder neglect scores in education level, nature of the institution, nature of employment, working years, daily working hours, days off per month, whether received pre-service training, whether received regular training after induction, whether worked with a license, level of qualification certificate, monthly salary and whether had social insurances (*p* < 0.05), but not in gender, age, residence, marital status, and number of older adults cared for per day (*p* > 0.05) (Table 2).

Specifically, among the personal factors, geriatric nursing assistants with primary school education or below scored lower on elder neglect than those with university or above (*p* = 0.009), with no differences between the other groups (*p* > 0.05). Contract workers scored lower on elder neglect than regular (*p* = 0.001) and temporary workers (*p* < 0.001), while there was no difference in scores between regular and temporary workers (*p* > 0.05). Interestingly, elder neglect scores were highest among geriatric nursing assistants with 3–5 years of work and lowest among those with 10 or more years of work (*p* < 0.001), and no differences were found between the other groups (*p* > 0.05). No licensed geriatric nursing assistants had lower scores for elder neglect (Median = 73; IQR: 64–78). Geriatric nursing assistants without qualifications scored lower than those with primary, intermediate, advanced, and qualified qualifications for elder neglect (*p* < 0.01), while those with primary qualifications scored lower than those with advanced qualifications (*p* = 0.002), and there were no differences between the other groups (*p* > 0.05).

In the organizational factor, lower elder neglect scores for staff working in public care institutions than those working in private care institutions (*p* < 0.001), and there were no differences between the other groups (*p* > 0.05). Regarding working and rest time, geriatric nursing assistants who worked 15–24 h per day had lower scores for elder neglect than those working 8–10 h per day and 11–14 h per day (*p* < 0.05), while there was no difference in scores between those working 8–10 h per day and those working 11–14 h per day (*p* > 0.05). Geriatric nursing assistants with no more than 2 days off per month had lower scores for elder neglect than those with 3–4 days off per month and ≥ 5 days off per month (*p* < 0.001), while there was no difference in scores between those with 3–4 days off per month and with ≥ 5 days off per month (*p* > 0.05). The elder neglect scores were lower for geriatric nursing assistants with no pre-service training (Median = 69.5; IQR: 61.25–72) and no regular training after induction (Median = 65; IQR: 61.5–71). Geriatric nursing assistants with a monthly salary of less than or equal to 1,900 RMB scored lower on elder neglect than those with a monthly salary of 1,901–3,000 RMB, 3,001–4,000 RMB, and 4,001–5,000 RMB (*p* < 0.001). There was no difference in scores between 1,901–3,000 RMB, 3,001–4,000 RMB, and 4,001–5,000 RMB monthly salaries (*p* > 0.05). Geriatric nursing assistants without social insurance scored even lower for elder neglect (Median = 73; IQR: 67–80) (Table 2).

### 3.4. GSES scores and PPS scores and correlation with the elder neglect

The median score on the GSES and PPS of geriatric nursing assistants were 28 (IQR: 25–32) and 40 (IQR: 37–43), respectively (Table 3). Self-efficacy scores (r_s_ = 0.322, *p* < 0.001) and proactive personality scores (r_s_ = 0.397, *p* < 0.001) showed a moderate positive correlation with total elder neglect scores for geriatric nursing assistants. This indicated that the higher the self-efficacy score, the higher the elder neglect score, and the higher the proactive personality score, the higher the elder neglect score. Besides, a weak positive correlation was found between the self-efficacy score and proactive personality score (r_s_ = 0.285, *p* < 0.001). It showed that the higher the self-efficacy score, the higher the proactive personality score.

### 3.5. Multiple linear regression analysis of factors influencing elder neglect by geriatric nursing assistants

Multiple collinearity diagnostics showed variance inflation factors of < 5 for all variables. Therefore, there is no multicollinearity in our data. The Durbin–Watson statistic used for autocorrelation was 1.639. The residual scatter plot confirmed the equality of variances and the residual histogram distribution showed normality. The stepwise regression analysis showed that qualification level, self-efficacy and proactive personality were personal factors significantly associated with elder neglect. Elder neglect scores were higher for geriatric nursing assistants with advanced qualifications than for those without qualifications (*β* = 0.08, *p* = 0.025). Additionally, the higher the score of self-efficacy, the lower the level of elder neglect by geriatric nursing assistants (*β* = 0.31, *p* < 0.001). Higher active personality scores were associated with lower levels of elder neglect (*β* = 0.18, *p* < 0.001).

In addition, in terms of organizational factors, monthly salary, monthly time off and training after induction were significant predictors of elder neglect. Geriatric nursing assistants earning ≤ 1,900 RMB per month had lower elder neglect scores than those earning 4,001–5,000 RMB per month (*β* = −0.26, *p* < 0.001). Elder neglect scores were higher for 3–4 days off per month (*β* = 0.19, *p* < 0.001) and ≥ 5 days off per month (*β* = 0.10, *p* = 0.014) than for ≤ 2 days off per month. The score of elder neglect for those without regular training after induction was lower than for those with regular training (*β* = −0.25, *p* < 0.001). Variables entering the final model explained 44.3% of the variation in elder neglect and were statistically significant (*F* = 47.691, *p* < 0.001) (Table 4).

**Table 4:** Multiple linear regression analysis of factors influencing elder neglect by geriatric nursing assistants (*n* = 412).

The independent variable	Unstandardized coefficient		Standardization coefficient	*t*	*p*-value	Co-linear statistics	
	*B*	Standard Error	*Beta*			Tolerances	VIF
Constant	44.887	3.181		14.109	< 0.001		
Personal factors							
Advanced qualification	3.147	1.403	0.084	2.243	0.025	0.968	1.033
Self-efficacy score	0.534	0.064	0.312	8.324	< 0.001	0.962	1.039
Proactive Personality Score	0.336	0.072	0.180	4.680	< 0.001	0.918	1.089
Organizational factors							
Monthly salary (RMB) ≤ 1,900	−5.058	0.813	−0.256	−6.220	< 0.001	0.799	1.252
Days off per month 3–4 days	3.486	0.753	0.192	4.628	< 0.001	0.787	1.270
Days off per month ≥ 5 days	2.545	1.031	0.101	2.469	0.014	0.802	1.247
No regular training after induction	−5.819	0.879	−0.253	−6.618	< 0.001	0.928	1.078

*R*^2^ = 0.452, adjusted *R*^2^ = 0.443, *F* = 47.691, *p*-value < 0.001.

This study obtained the level of elder neglect among geriatric nursing assistants in Chinese nursing homes and identified personal and organizational factors that influence elder neglect. In addition, the results confirmed the association of self-efficacy and proactive personality with elder neglect.

## 4. Discussion

This cross-sectional study aimed to identify the current situation and the influencing factors of elder neglect among geriatric nursing assistants. The results were discussed in light of existing studies on this topic in the literature. Determining the factors influencing elder neglect by geriatric nursing assistants in nursing homes is critical to optimizing senior services, promoting the health of older adults, improving their quality of life, and reducing their healthcare expenditures (e.g., hospitalization expenditures due to physical or psychological injuries caused by elder neglect).

### 4.1. The current state of geriatric nursing assistant elder neglect

The median total score of elder neglect for geriatric nursing assistants was 74 out of 85, indicating that geriatric nursing assistants were at a low level of elder neglect, which was consistent with the results of a previous study (20). This may be because most of the geriatric nursing assistants in this survey have received pre-service training and regular post-induction training. Geriatric nursing assistants could possess basic nursing skills (27) and have the capability to enhance their level of service. On the other hand, caution is needed when interpreting the estimates from staff self-report (4). Negligent geriatric nursing assistants may conceal, dilute or even deny the existence of elder neglect for various reasons (28), resulting in reporting bias. Therefore, the data may be underestimated. However, elder neglect by geriatric nursing assistants is objective. The study found that some geriatric nursing assistants in the surveyed areas did not focus on the wishes of older adults and did not help them with needed health care. As a result, some older adults lack glasses, hearing aids, dentures, and other assistive devices. The occurrences agree with the existing literature (29). This emphasizes the need to further improve the quality of training for geriatric nursing assistants and to focus on guiding them to adopt a people-oriented service philosophy. Modules on how to communicate effectively with older adults and better understand their needs should be included in the training. In addition, quality control of geriatric care should not be overlooked. Nursing home management should establish a sound monitoring mechanism to regularly inspect and evaluate the work of geriatric nursing assistants, and at the same time, the results of the evaluation should be incorporated into the system of rewards and punishments for geriatric nursing assistants.

In addition, in this study, physical and medical neglect scores were higher, while psychological and economic neglect scores were lower. It indicated that the geriatric nursing assistants focused more on the physical and medical care of older adults, but less on their psychological and economic needs. The reasons may be related to the geriatric nursing assistants’ work characteristics and institutional facilities. The majority of the geriatric nursing assistants (82.3%) in this study receive regular training in the daily care of older adults after their induction. Examples include daily living care, common symptom care, and safety care. This allows geriatric nursing assistants to acquire knowledge and skills in daily care and medication health care, both of which have the potential to reduce the occurrence of physical and medical neglect (30). Psychological neglect may result from the fact that geriatric nursing assistants are required to care for multiple older adults simultaneously and are overwhelmed with work, making it difficult to find time to meet the emotional communication needs of older adults (31). Furthermore, nursing homes are not well equipped with hardware facilities to meet the need for privacy protection. For example, beds in some nursing homes are largely devoid of bed curtains, which leaves geriatric nursing assistants without bed curtains to cover them when handling older adults’ urination and defecation. This belongs to the category of psychological neglect. The lowest score is economic neglect. Some of the nursing homes in this survey stipulate that conscious older adults can keep their possessions by themselves, but geriatric nursing assistants may not be comfortable asking older adults too much about whether they have discretionary possessions (e.g., cash, valuable jewelry, etc.) because the status of their possessions is a sensitive topic. This prevents geriatric nursing assistants from understanding the financial situation of older adults and providing timely assistance to avoid financial loss (e.g., loss of cash, etc.). Therefore, in order to cope with the problem of elder neglect, it is crucial to strengthen publicity and education. According to the work characteristics of geriatric nursing assistants, web-based teaching can be utilized to provide geriatric nursing assistants with convenient learning pathways so that they can learn anytime and anywhere. In addition, offline learning seminars and outbound training can be organized regularly to deepen their knowledge of psychological care and enhance their awareness of and attention to the safety of the property of older adults. In addition, improving hardware facilities to meet the privacy needs of older adults should also be brought to the attention of nursing home managers.

### 4.2. Personal factors in elder neglect by geriatric nursing assistants

Our study found that the total elder neglect score was positively correlated with the self-efficacy score and that self-efficacy was an influential factor in elder neglect. In other words, the higher the geriatric nursing assistants’ self-efficacy, the lower their levels of elder neglect. It suggests that hypothesis 2 holds. This is similar to the results of a previous study (32). Self-efficacy, as one of the important sources of psychological capital for individuals, reflects the level of confidence of geriatric nursing assistants in achieving goals and accomplishing work. When geriatric nursing assistants have high levels of self-efficacy, they are able to face challenges in their work with greater confidence (33, 34) and thus are more attentive and responsive to the needs of older adults. The source of this confidence may be related to their past successes, ongoing career development opportunities, and team support (35). In contrast, geriatric nursing assistants with low self-efficacy will feel uneasy and anxious in the face of challenges at work, which may lead them to neglect the needs of older adults. In addition, geriatric nursing assistants with high self-efficacy are more likely to develop positive relationships with older adults (36), which contributes to their sensitivity to the needs of older adults. Therefore, the self-efficacy of geriatric nursing assistants provides ideas for reducing elder neglect. Policymakers should provide clear and viable career advancement opportunities to ensure that geriatric nursing assistants have access to the necessary support. Nursing home administrators should create a supportive work environment that encourages communication and collaboration among geriatric nursing assistants. Geriatric nursing assistants should actively participate in training where they can learn from the past successes of other geriatric nursing assistants.

In the present study, a positive connection was found between the total score of elder neglect and the score of proactive personality, revealing that when geriatric nursing assistants’ level of proactivity increased, their level of elder neglect decreased. This indicates that hypothesis 2 holds. Highly proactive employees have positive attitudes and behaviors. Individuals with high levels of proactivity can actively cope with stressful job demands to prevent burnout (37). Burnout is a syndrome caused by chronic work stress among employees. It is characterized by loss of energy, feeling tired, increasing psychological distance from work, work-related negativity, and decreasing professional efficacy (38). Natan et al. (39) found that the greater the level of employee burnout, the higher their risk of elder abuse and neglect. Therefore, the proactive personality of geriatric nursing assistants may help reduce burnout and then decrease the level of elder neglect. However, previous studies have rarely used burnout to explain the connection between geriatric nursing assistants’ proactive personalities and the risk of elder neglect. This hypothesis needs to be further verified in future studies. The result suggests that during the recruitment process, nursing home administrators should take care in selecting geriatric nursing assistants with proactive personality. This can be selected through questionnaire tests, interviews and observation during the internship period. Meanwhile, in training, attention should be paid to cultivating the initiative and service consciousness of geriatric nursing assistants, encouraging them to take the initiative in caring for older adults, discovering their needs and actively solving problems.

From our findings, the level of qualification obtained by geriatric nursing assistants is an influential factor in elder neglect. Geriatric nursing assistants with advanced levels of qualification have lower levels of elder neglect. This is consistent with previous research findings (40). The reasons may be manifold. The qualification requires geriatric nursing assistants with a certain level of work experience to participate in the caregiving training course and pass the relevant exams (41). Thus, geriatric nursing assistants with advanced qualifications have a higher level of theoretical knowledge and clinical care skills, meet national standards, and have the ability to respond professionally, which helps reduce the level of elder neglect. In addition, a Norwegian study showed that geriatric nursing assistants with a high propensity to leave were more likely to engage in elder neglect (7). Highly qualified geriatric nursing assistants generally exhibit a strong sense of professional identity and accomplishment, and are less likely to leave their job, which reduces the risk of elder neglect. Accordingly, in promoting geriatric nursing assistants, priority should be given to those candidates with advanced qualifications to ensure that older adults receive better quality care. In addition, the level of qualifications of geriatric nursing assistants should be taken into account in the formulation of the remuneration system, so as to provide incentives for them to actively participate in training and take the initiative to improve their nursing skills.

### 4.3. Organizational factors in elder neglect by geriatric nursing assistants

In addition, this study considered the impact of organizational factors on geriatric nursing assistants’ elder neglect. The regression analysis shows that hypothesis 1 seems to hold. In this study, 27.2% of geriatric nursing assistants earn less than or equal to RMB 1,900 per month. It is necessary to point out that RMB 1,900 is the minimum monthly salary standard in the investigated area. As you can see, some of the geriatric nursing assistants are extremely underpaid. This study showed that a monthly salary of less than or equal to RMB 1,900 would increase the risk of elder neglect. This may be related to symptoms of psychological distress caused by economic and psychological stress (42) among low-wage geriatric nursing assistants. Symptoms of psychological distress may lead to elder neglect (7). The government should provide appropriate assistance to help geriatric nursing assistants to solve their financial difficulties. For example, benefits such as housing subsidies and transportation subsidies can be provided to alleviate the economic burden of geriatric nursing assistants. The wage system for geriatric nursing assistants should also be reassessed to ensure that all nursing homes comply with the regional minimum wage. Nursing home administrators should implement a performance incentive system that provides additional incentives to high-performing geriatric nursing assistants based on feedback from older adults and their families, quality of care, and other relevant evaluation criteria. This will help increase geriatric nursing assistants’ job satisfaction and enable them to provide better care to older adults (43), thereby reducing the risk of elder neglect.

Our results showed that the total elder neglect score was positively associated with three or more days off per month. This shows that geriatric nursing assistants with three or more days off per month have a lower level of elder neglect than those with less than 3 days off per month. Rest is a crucial factor in maintaining the job satisfaction of workers (44). It provides individuals with the opportunity to release accumulated stress and rejuvenate their bodies and minds, thus enabling them to maintain a positive attitude toward work. Otherwise, poor attitudes of geriatric nursing assistants can increase the level of elder neglect in nursing homes (7). However, in China, geriatric nursing assistants do not have the benefit of national holiday breaks and are expected to work regular and busier shifts during festivals and holidays (31). It is therefore imperative to provide adequate rest and improve the working environment of geriatric nursing assistants. This will not only improve the job satisfaction of geriatric nursing assistants, but will also have a positive impact on protecting the health and well-being of older adults. We call on the community to pay attention to this issue and recommend that the government introduce relevant regulations to ensure the right of geriatric nursing assistants to take holidays off, and at the same time join hands with universities to train specialized talents in elder care and expand the team of geriatric nursing assistants, so as to alleviate the per capita workload and effectively implement a flexible work system.

Furthermore, results of the current study indicate that geriatric nursing assistants who receive regular training after induction have a lower level of elder neglect. In the study, most geriatric nursing assistants are qualified through pre-service training and receive regular training after their employment. Regular post-induction training is important for geriatric nursing assistants. Without regular training after induction, employees could not update their theoretical knowledge and professional skills (45). Resident aggression and care-related conflicts are factors associated with elder neglect (7). Geriatric nursing assistants, who have received regular training after induction and may have better caregiving skills, are relatively better able to cope with resident aggression and reduce the occurrence of caregiving-related conflicts. In this case, geriatric nursing assistants are likely to be able to provide optimal care to older adults promptly, which may lead to reducing the level of elder neglect. Therefore, future training needs to be strengthened. Taking into account the current situation that the literacy levels of geriatric nursing assistants are not homogeneous (32), a hierarchical training approach may be beneficial in the curriculum design. Specifically, for newly employed geriatric nursing assistants with a limited academic background, emphasis should be placed on life care knowledge with a corresponding reduction in theoretical instruction. After geriatric nursing assistants have gained some experience in elder care or have developed a need for further training, they can strengthen training in the care of common chronic diseases of older adults, first-aid care, psychological care and other content, and strengthen theoretical guidance on the basis of improving their operational abilities so that they can take on the care of older adults with more complex medical conditions.

### 4.4. Study limitations

There are some limitations to the survey. Firstly, our study was conducted in a selection of Chinese nursing homes using a convenience sampling method, which may lead to potential selection bias and limit the generalizability of the findings. And due to geographic and cultural differences, it may lead to a lack of representativeness in the results. Therefore, it is necessary to conduct multicenter studies using random sampling methods in the future. Secondly, elder neglect is a sensitive topic. The sensitivity of the topic may lead participants to answer questions untruthfully out of concern, or fear, or to avoid social judgment. This may result in information bias in the study and affect the truthfulness and accuracy of the findings. Future studies can obtain information from multiple perspectives, such as managers and older adults, to ensure the reliability of the data. Finally, this study utilized a cross-sectional design, obtaining data that only presents a snapshot of the current situation, not allowing for causal inferences. Longitudinal studies can follow the same group of participants over several time points, offering greater potential to investigate the causal mechanisms underlying the observed associations. Therefore, longitudinal studies could be conducted in the future to more accurately analyze influencing factors.

## 5. Conclusion

This study found low level elder neglect in the study sample. Additionally, our findings indicated the organizational factors and personal characteristics of geriatric nursing assistants were important factors of elder neglect. In organizational factors, monthly salary, monthly days off, and regular training after induction are associated with elder neglect. In personal factors, having an advanced qualification reduces the level of elder neglect. In addition, this study confirms that self-efficacy and the proactive personality of geriatric nursing assistants play a positive role in reducing elder neglect in nursing homes. In the absence of other studies of geriatric nursing assistants, our study provides important information to proactively address the aging population and mitigate elder neglect. Further prospective studies are warranted. In order to alleviate the phenomenon of elder neglect, relevant policies should be formulated to develop the elder service industry by strengthening the nursing workforce and promoting the professionalization of geriatric nursing assistants.

## Data availability statement

The raw data supporting the conclusions of this article will be made available by the authors, without undue reservation.

## Ethics statement

The studies involving humans were approved by Medical Ethics Committee of Tianjin University of Traditional Chinese Medicine. The studies were conducted in accordance with the local legislation and institutional requirements. The participants provided their written informed consent to participate in this study.

## Author contributions

JW: Writing – review & editing, Conceptualization, Writing – original draft. ZY: Conceptualization, Writing – original draft, Writing – review & editing. YL: Writing – original draft, Writing – review & editing. RM: Writing – review & editing, Formal analysis, Investigation. LZ: Investigation, Writing – review & editing. YD: Investigation, Writing – review & editing. HD: Writing – review & editing, Formal analysis.

## Abbreviations

IQR: Interquartile range
WHO: World Health Organization
GSES: General Self-Efficacy Scale
PPS: Proactive Personality Scale
VIF: Variance Inflation Factor
