# Supplementary material for: Status and influencing factors of elder neglect by geriatric nursing assistants in Chinese nursing homes: a cross-sectional survey
Source: Front Med (Lausanne). 2023 Oct 25;10:1273289. doi: 10.3389/fmed.2023.1273289 (PMC10634532; doi:10.3389/fmed.2023.1273289)
Supplement: Supplementary file 1 [file Table_1.docx]

***Supplementary Material***

Status and influencing factors of elder neglect by geriatric nursing assistants in Chinese nursing homes: a cross-sectional survey

**Jing Wang^1^**^†^**, Zhihua Yang^1^**^†^**, Ya Li^1^, Ruijuan Ma^1^, Liping Zhang^1^, Yage Du^2^, Haoying Dou^1*^**

**^†^These authors share first authorship.**

*** Correspondence:** Haoying Dou: [douhaoying11@126.com](mailto:douhaoying11@126.com)

1. **Supplementary Tables**

**Table 1.** STROBE Statement—checklist of items that should be included in reports of observational studies

|  | **Item No.** | **Recommendation** | **Page No.** | **Relevant text from manuscript** |
| --- | --- | --- | --- | --- |
| **Title and abstract** | 1 | (*a*) Indicate the study’s design with a commonly used term in the title or the abstract | 1 | Cross-sectional survey |
|  |  | (*b*) Provide in the abstract an informative and balanced summary of what was done and what was found | 1 | From the perspective of geriatric nursing assistants, this study aims to explore the current situation and influencing factors of elder neglect in Chinese nursing homes. |
| **Introduction** |  |  |  |  |
| Background/rationale | 2 | Explain the scientific background and rationale for the investigation being reported | 2 | The background of the study included elder neglect definition, manifestations, adverse outcomes, prevalence, and risk factors. The rationale for the study included nursing home characteristics, self-efficacy, and proactive personality in relation to elder neglect. |
| Objectives | 3 | State specific objectives, including any prespecified hypotheses | 3 | This study aims to investigate the level of elder neglect in Chinese nursing homes and its association with geriatric nursing assistants' personal and organizational factors, such as compensation, training, supervision, and working conditions (H1). Additionally, we hypothesize that high levels of self-efficacy and a proactive personality among geriatric nursing assistants will reduce the level of elder neglect (H2). |
| **Methods** |  |  |  |  |
| Study design | 4 | Present key elements of study design early in the paper | 3 | This survey used a cross-sectional design. |
| Setting | 5 | Describe the setting, locations, and relevant dates, including periods of recruitment, exposure, follow-up, and data collection | 3 | The geriatric nursing assistants were recruited from 50 nursing homes registered with the Civil Affairs Bureau in Henan, Hebei, and Shandong provinces, China. The information was gathered between July and September 2020. |
| Participants | 6 | (*a*) *Cohort study*—Give the eligibility criteria, and the sources and methods of selection of participants. Describe methods of follow-up  *Case-control study*—Give the eligibility criteria, and the sources and methods of case ascertainment and control selection. Give the rationale for the choice of cases and controls  *Cross-sectional study*—Give the eligibility criteria, and the sources and methods of selection of participants | 3 | The inclusion criteria were: (1) directly responsible for caring for older adults, (2) caring for an older adult in a nursing home for at least 3 months, (3) having the basic reading, writing, or understanding ability, and (4) can voluntarily provide informed consent. The exclusion criteria were: (1) not on duty during the study period. Geriatric nursing assistants were recruited from nursing homes in Henan, Hebei, and Shandong provinces, China. The researchers obtained the contact information of the nursing homes in the three regions from the official website of the Ministry of Civil Affairs and then obtained the consent and support of 50 nursing home directors through telephone or email contact. |
|  |  | (*b*) *Cohort study*—For matched studies, give matching criteria and number of exposed and unexposed  *Case-control study*—For matched studies, give matching criteria and the number of controls per case | N/A |  |
| Variables | 7 | Clearly define all outcomes, exposures, predictors, potential confounders, and effect modifiers.  Give diagnostic criteria, if applicable | 3, 4 | The geriatric nursing assistant's gender, age, household location, marital status, and education level, license, years of experience, salary, nature of employment, number of elderlies to care per day, working hours, general self-efficacy, proactive personality. |
| Data sources/ measurement | 8* | For each variable of interest, give sources of data and details of methods of assessment (measurement). Describe comparability of assessment methods if there is more than one group | 3, 4 | Demographic Questionnaire, Elder Neglect Scale for Geriatric Nursing Assistants, General Self-Efficacy Scale (GSES), and Proactive Personality Scale (PPS). |
| Bias | 9 | Describe any efforts to address potential sources of bias | 3 | Before the survey, all researchers received uniform training to ensure consistency of study results. The researchers were familiar with the content of the questionnaires and standardized the instructions' terminology after training to avoid vague and misleading statements. Additionally, appropriate quality control measures are taken to ensure the data's authenticity, reliability and completeness. First, the researchers selected elderly caregivers according to strict criteria and distributed the questionnaires in several nursing homes in several regions to minimize selection bias caused by convenience sampling. Second, given the sensitivity of the research questions, the anonymity and confidentiality of the survey were declared to participants before completing the questionnaire to eliminate their concerns. In addition, it was necessary to ensure that no one else was present during the investigation to minimize self-reporting bias. Finally, the questionnaires were distributed and collected on the spot, and participants were asked to fill in any missing entries. |
| Study size | 10 | Explain how the study size was arrived at | 3 | The sample size should be 5-10 times the number of variables, according to the Kendall sample estimate method. The number of variables included in this survey was 37. As a result, we determined that the sample size for this study should be 185-370. In addition, we statistically considered that there may be a 20% questionnaire failure rate, and 232-463 questionnaires should be finally distributed. |
| Quantitative  variables | 11 | Explain how quantitative variables were handled in the analyses. If applicable, describe which groupings were chosen and why | 4 | To determine if the data had a normal distribution, the Shapiro-Wilk test was utilized, which showed that the data was nonnormally distributed. Therefore, frequencies and percentages were used for descriptive statistics for categorical data and median and interquartile range (IQR) for continuous variables. |
| Statistical  methods | 12 | (*a*) Describe all statistical methods, including those used to control for confounding | 4 | The Mann-Whitney U test was used for dichotomous variables, and the Kruskal-Wallis test was used for multicategorical variables to determine if there was a significant relationship between the distributions of elder neglect by geriatric nursing assistants' characteristics. Bonferroni was used for post-hoc comparisons. Correlation analysis was performed using Spearman's test to evaluate the association of geriatric nursing assistants' proactive personality, self-efficacy, and elder neglect. To control for confounding variables, variables showing statistically significant differences (P < 0.05) in the results of univariate and correlation analyses were included in stepwise multiple linear regression analyses to identify factors influencing elder neglect. |
|  |  | (*b*) Describe any methods used to examine subgroups and interactions | N/A |  |
|  |  | (*c*) Explain how missing data were addressed | 4 | If the entry missing value or invalid answers were greater than or equal to 5%, the questionnaire was excluded. If the missing value of entries was within 5%, the mean value method was used to estimate the average of all response units. |
|  |  | (*d*) *Cohort study*—If applicable, explain how loss to follow-up was addressed  *Case-control study*—If applicable, explain how matching of cases and controls was addressed  *Cross-sectional study*—If applicable, describe analytical methods taking account of sampling strategy | N/A |  |
|  |  | (*e*) Describe any sensitivity analyses | N/A |  |
| **Results** |  |  |  |  |
| Participants | 13* | (a) Report numbers of individuals at each stage of study—eg numbers potentially eligible, examined for eligibility, confirmed eligible, included in the study, completing follow-up, and analysed | 4 | A total of 449 questionnaires were distributed, excluding 20 containing incomplete responses and 17 with invalid answers, 412 valid questionnaires were received, with a valid recovery rate of 91.8%. |
|  |  | (b) Give reasons for non-participation at each stage | 4 | A total of 449 questionnaires were distributed, excluding 20 containing incomplete responses and 17 with invalid answers, 412 valid questionnaires were received, with a valid recovery rate of 91.8%. |
|  |  | (c) Consider use of a flow diagram | N/A |  |
| Descriptive data | 14* | (a) Give characteristics of study participants (eg demographic, clinical, social) and information on exposures and potential confounders | 4 | More demographic information is shown in Table 2. |
|  |  | (b) Indicate number of participants with missing data for each variable of interest | N/A |  |
|  |  | (c) Cohort study—Summarise follow-up time (eg, average and total amount) | N/A |  |
| Outcome data | 15* | Cohort study—Report numbers of outcome events or summary measures over time | N/A |  |
|  |  | Case-control study—Report numbers in each exposure category, or summary measures of exposure | N/A |  |
|  |  | Cross-sectional study—Report numbers of outcome events or summary measures | 6, 7 | Characteristics of the Elder Neglect Scale for Geriatric Nursing Assistants scores are demonstrated in detail in Table 3. The results of the univariate analysis of the elder neglect (Table 2). The GSES scores and PPS scores (Table 3). The results of multiple linear regression analysis of the elder neglect (Table 4). |
| Main results | 16 | (a) Give unadjusted estimates and, if applicable, confounder-adjusted estimates and their precision (eg, 95% confidence interval). Make clear which confounders were adjusted for and why they were included | 7 | Table 4 shows the multivariate analysis of factors that can predict elder neglect. |
|  |  | (b) Report category boundaries when continuous variables were categorized | 5, 6 | Table 2 shows the category boundaries. Age category: ≤ 30 years old, 31-40 years old, … |
|  |  | (c) If relevant, consider translating estimates of relative risk into absolute risk for a meaningful time period | N/A |  |
| Other analyses | 17 | Report other analyses done—eg analyses of subgroups and interactions, and sensitivity analyses | N/A |  |
| **Discussion** |  |  |  |  |
| Key results | 18 | Summarise key results with reference to study objectives | 7, 8 | The median total score of elder neglect for geriatric nursing assistants was 74 out of 85, indicating that geriatric nursing assistants were at a low level of elder neglect, which was consistent with the results of a previous study. |
| Limitations | 19 | Discuss limitations of the study, taking into account sources of potential bias or imprecision. Discuss both direction and magnitude of any potential bias | 10 | Firstly, our study was conducted in a selection of Chinese nursing homes using a convenience sampling method, which may lead to potential selection bias and limit the generalizability of the findings. Due to geographic and cultural differences, it may lead to a lack of representativeness in the results. Secondly, elder neglect is a sensitive topic. The sensitivity of the topic may lead participants to answer questions untruthfully out of concern, or fear, or to avoid social judgment. This may result in information bias in the study and affect the truthfulness and accuracy of the findings. Finally, this study utilized a cross-sectional design, obtaining data that only presents a snapshot of the current situation, not allowing for causal inferences. |
| Interpretation | 20 | Give a cautious overall interpretation of results considering objectives, limitations, multiplicity of  analyses, results from similar studies, and other relevant evidence | 7, 8, 9, 10 | This cross-sectional study aimed to identify the current situation and the influencing factors of elder neglect among geriatric nursing assistants. In addition, this study considered the impact of personal and organizational factors of geriatric nursing assistants on elder neglect. |
| Generalisability | 21 | Discuss the generalisability (external validity) of the study results | 10 | Our study was conducted in a selection of Chinese nursing homes using a convenience sampling method, which may lead to potential selection bias and limit the generalizability of the findings. And due to geographic and cultural differences, it may lead to a lack of representativeness in the results. |
| **Other information** |  |  |  |  |
| Funding | 22 | Give the source of funding and the role of the funders for the present study and, if applicable, for the original study on which the present article is based | N/A |  |

*Give information separately for cases and controls in case-control studies and, if applicable, for exposed and unexposed groups in cohort and cross-sectional studies.

**Note:** An Explanation and Elaboration article discusses each checklist item and gives methodological background and published examples of transparent reporting. The STROBE checklist is best used in conjunction with this article (freely available on the Web sites of PLoS Medicine at [http://www.plosmedicine.org/,](http://www.plosmedicine.org/) Annals of Internal Medicine at [http://www.annals.org/,](http://www.annals.org/) and Epidemiology at [http://www.epidem.com/).](http://www.epidem.com/)) Information on the STROBE Initiative is available at [www.strobe-statement.org.](http://www.strobe-statement.org/)
